# Comparative Analysis of Transradial and Transfemoral Approaches in Transarterial Radioembolization for Liver Tumors: A Systematic Review and Meta-Analysis

**DOI:** 10.1007/s00270-024-03865-z

**Published:** 2024-10-07

**Authors:** Hatem Abdelmoneim Eldeeb, Mahmoud Shaaban Abdelgalil, Asem Ahmed Ghalwash, Asmaa Elganady, Ruaa Mustafa Qafesha, Ibraheem M. alkhawaldeh, Mahmoud Diaa Hindawi, Jaber H. Jaradat, Shabaan Mohamed Abduljalil, Hussien Ahmed H. Abdelgawad

**Affiliations:** 1 Faculty of Medicine, Alazhar University, Cairo, Egypt; 2 Faculty of Medicine, Ain-Shams University, 359 Abd Allah Nadim Street, Cairo, Egypt; 3 Faculty of Medicine, Al Azhar University, Cairo, Egypt; 4 Medical Research Group of Egypt, Negida Academy, Arlington, MA USA; 5 Faculty of Medicine, Alexandria University, Alexandria, Egypt; 6 Faculty of Medicine, Al-Quds University, Jerusalem, Palestine; 7 Faculty of Medicine, Mutah University, Al-Karak, Jordan; 8 Eradah Complex and Mental Health Hospital, Najran, Kingdom of Saudi Arabia; 9 Department of Child Health, University of Arizona College of Medicine, Phoenix, AZ USA

**Keywords:** Transarterial radioembolization, Transradial, Transfemoral, Liver tumors, Meta-analysis

## Abstract

**Purpose:**

Transarterial radioembolization (TARE) is a minimally invasive therapy combining embolization and radiation for cancer treatment. This meta-analysis compares radiation exposure, quality of life, and safety of the transradial (TRA) versus transfemoral (TFA) approaches in TARE for liver tumors.

**Materials and Methods:**

We searched PubMed, SCOPUS, Cochrane, EMBASE, and Web of Science for studies comparing TRA versus TFA in TARE for liver tumors. Our primary outcomes focused on various measures of patient radiation exposure, including procedure time, fluoroscopy time, air kerma, and dose-area product (DAP). For secondary outcomes, we evaluated safety parameters, such as overall pain experienced during the procedure, pain in the recovery room post-procedure, the incidence of adverse events, and the impact on quality of life. Study quality was assessed using Cochrane’s ROB 2 tool for RCTs and the Newcastle–Ottawa scale for observational studies. Data analysis was conducted with REVMAN 5.4.1 software.

**Results:**

Six studies, comprising one RCT and five cohort studies with 1,209 patients, underwent comprehensive analysis. The aggregated findings revealed a significant reduction in procedure duration associated with TRA (MD =− 6.30, 95% CI [− 9.88, − 2.73], *P* = 0.005). However, no statistically significant differences were found between TRA and TFA groups concerning fluoroscopy time, recovery time, air kerma, DAP, pain in the recovery room, overall pain during the procedure, quality of life measuring mental health and physical function or adverse events.

**Conclusion:**

TRA and TFA showed comparable results in TARE for liver tumors, but TRA offered a shorter procedure time. Further RCTs with larger samples are needed to confirm these findings. Future studies should assess long-term efficacy for a more complete evaluation.

**Graphical Abstract:**

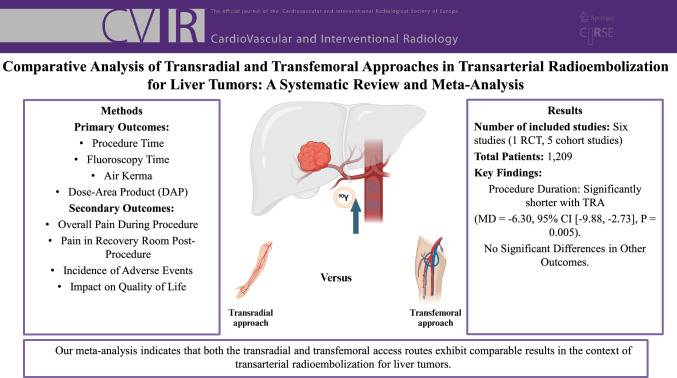

**Supplementary Information:**

The online version contains supplementary material available at 10.1007/s00270-024-03865-z.

## Introduction

Transarterial radioembolization (TARE), also known as selective internal radiation therapy, is a minimally invasive procedure used by interventional radiologists to treat liver tumors and metastases. This treatment involves the delivery of radioactive microspheres directly into the hepatic artery. These microspheres locally irradiate the liver tumors, providing targeted radiation therapy while minimizing exposure to surrounding healthy tissue [1, 2].

Traditionally, transfemoral artery access (TFA) has been the mainstay for hepatic radioembolization. However, transradial artery access (TRA) offers a promising alternative, with its superficial, compressible nature reducing entry site complications and improving post-procedure comfort. Its use has surged in cardiac procedures due to these benefits [3, 4]. Recent evidence suggests that TRA may offer advantages over TFA in certain cases. A meta-analysis by Senguttuvan et al. found that TRA reduced adverse events like bleeding and vascular complications in STEMI patients without compromising outcomes like re-infarction or stroke. However, concerns remain about technical difficulties, longer procedures, and increased radiation exposure [5].

Despite TRA is commonly used in coronary interventions, these drawbacks may contribute to the reluctance of interventional radiologists to adopt TRA for systemic circulation interventions [3]. However, recent trials in transarterial radioembolization for liver tumors show that TRA offers benefits such as earlier ambulation, better patient comfort, reduced bleeding risks, and faster recovery compared to TFA. Nonetheless, concerns persist regarding longer fluoroscopy durations, increased radiation exposure to patients, and higher rates of access failure associated with TRA [3, 6].

Given the conflicting results from previous trials [3, 6, 7], this meta-analysis aims to provide a comprehensive comparison of patient radiation exposure, quality of life, and safety between the TRA and TFA approaches for the treatment of hepatic tumors using transarterial radioembolization.

## Methods

This meta-analysis was registered at the International Prospective Register of Systematic Reviews (PROSPERO) under registration number CRD42023475678 and adhered to the Preferred Reporting Items for Systematic Reviews and Meta-Analyses (PRISMA) guidelines [8] and Cochrane Handbook [9].

### Literature Search

We conducted a search on October 11, 2023, utilizing PubMed, Cochrane, Scopus, Web of Science, and Embase databases. Our search employed the key terms ‘transarterial radioembolization,’ ‘Transradial,’ ‘transfemoral,’ and ‘liver tumors.’ The detailed search strategy is available in the supplementary file figure file.

### Eligibility Criteria and Study Selection

Eligible studies were randomized controlled trials (RCTs) and cohort studies comparing the transradial versus the transfemoral approaches in liver tumor patients treated by TARE. We excluded case reports, cross-sectional studies, editorials, reviews, and studies not reporting primary outcomes. Only English-language publications were considered.

We used Rayyan.ai to manage and remove duplicate studies from all databases [10]. Two independent authors then screened the remaining titles and abstracts for eligibility. Eligible studies underwent full-text assessment, and if the full text was unavailable, we contacted the authors via email. Discrepancies were resolved by consensus or with a third reviewer. We also manually reviewed reference lists and performed backward citation analysis to ensure a comprehensive review.

### Data Extraction and Study Outcomes

Data extraction was independently performed by two reviewers using a standardized extraction sheet. The extracted data included the study characteristics (author, year, country, and study design), participant characteristics (sample size, age, sex, and comorbidities), and outcomes of interest. Our primary outcomes focused on various measures of patient radiation exposure, including procedure time, fluoroscopy time, air kerma, and dose-area product (DAP). For secondary outcomes, we evaluated safety parameters, such as overall pain experienced during the procedure, pain in the recovery room post-procedure, the incidence of adverse events, and the impact on quality of life.

### Quality Assessment and Risk of *Bias*

Two authors independently evaluated the quality and risk of bias of RCTs using the Cochrane RoB2 tool [11] and cohort studies using the Newcastle–Ottawa scale (NOS) [12]. Disagreements were resolved by consensus or a third reviewer.

### Data Synthesis and Statistical Analysis

We used Review Manager 5.4.1 for statistical analysis, applying mean difference (MD) for continuous data and risk ratios (RR) for dichotomous data. A *P*-value < 0.05 was considered significant. Initially using a fixed effects model, we switched to a random effects model upon detecting heterogeneity and performed a leave-one-out test. Heterogeneity was assessed with I2 (> 50%) and *P*-values (< 0.1) [13].

## Results

### Literature Search Results and Characteristics of the Included Studies

**Fig. 1:**
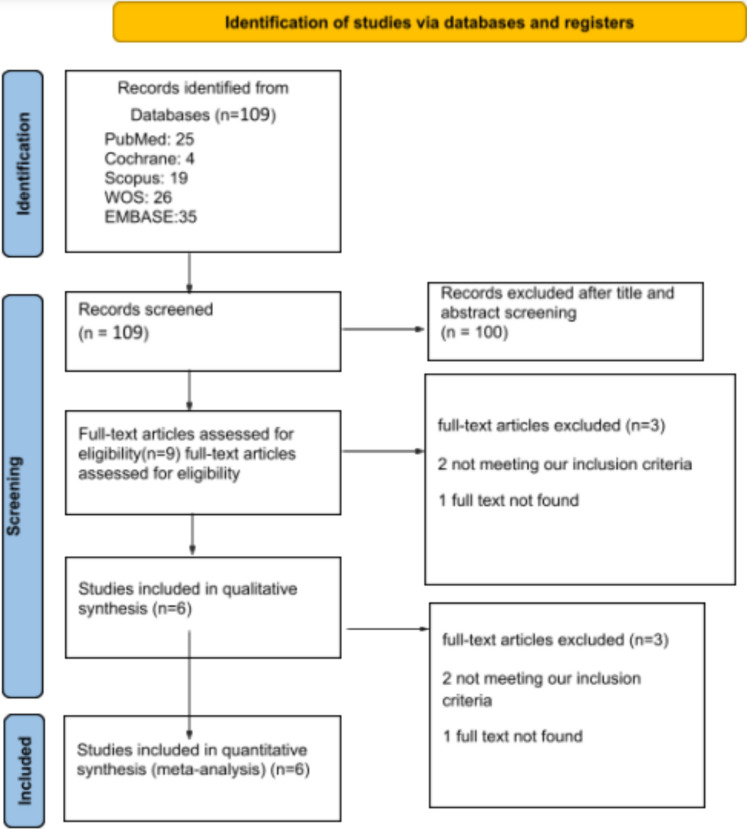
Preferred reporting items for systematic reviews and meta-analyses (PRISMA)

The search identified 109 studies, from which we included five retrospective cohorts [3, 6, 7, 14, 15] and one RCT [16] (Fig. 1). Our analysis covered 1,209 patients, with 632 undergoing TRA and 577 TFA. The TRA group had a mean age of 62.5 years, compared to 63.3 years for the TFA group. The studies were conducted in the USA (*N* = 5) [3, 6, 7, 14, 16] and Turkey (*N* = 1) [15], with follow-up ranging from 6 months to 5 years. Most studies used the left radial artery for TRA access and the right common femoral artery for TFA access, except for Bela Kis et al. [3], which utilized either the left or right radial artery for TRA and Yakupoglu et al. [15], which used either the left or right common femoral artery for TFA. Summaries of the studies are detailed in Tables 1 and 2**.**

**Table 1:** A summary of the included studies

Study Id	Country, site involved	Type of the study	Sample size of each group	TRA approach	TFA approach	Inclusion criteria	Cancer type	mean dose of GBq (mGy) of 90Y	90Y microsphere type	Study duration	Age (years) mean ± SD	Gender (male) *N*
Loewenstern 2018	USA	Retrospective cohort	After PSM; TRA 302/TFA 302	Left radial artery	Right common femoral artery	Patients who underwent a TARE procedure	HCC/Metastasis/other cancers	TRA = 323 mGy TFA = 248.46	Thera sphere/SIR Sphere	4 years (2013–2017)	TRA = 65.8 ± 10.3 TFA = 65.8 ± 10.6	TRA = 222 TFA = 221
Pedersoli 2023	USA	Retrospective cohort	TRA = 10 TFA = 12	Left radial artery	Right common femoral artery	Patients who underwent Y90 hepatic radioembolization	Liver Tumors	NA	Glass microspheres and labeled resin microspheres	1 year (2017–2018)	TRA = 55 TFA = 59	TRA = 5 TFA = 7
Bela Kis 2016	USA	Retrospective cohort	TRA = 27 TFA = 23	Left or right radial artery	Right common femoral artery	Patients who underwent Y90 hepatic radioembolization	Liver Tumors	TRA = 597.8 TFA 302.8	Glass microspheres	1.5 years (2014–2015)	TRA = 67 ± 10.9 TFA = 63 ± 13.9	TRA = 15 TFA = 15
Yakupog 2023	Turkey	Retrospective cohort	TRA = 75 TFA = 147	Left radial artery	Left or right common femoral artery	Underwent Y90 hepatic radioembolization either (TRA or TFA)	HCC	TRA = 545 mGy TFA = 893 mGy	(Y90) glass microspheres	5 years (2017–2022)	TRA = 59.4 ± 14.8 TFA = 60.9 ± 13.5	TRA = 39 TFA = 96
Ghosh 2021	USA	Retrospective cohort	TRA = 188 TFA = 63	Left radial artery	Right common femoral artery	Underwent Y90 hepatic radioembolization either (TRA or TFA)	HCC	TRA = 880.2 mGy TFA = 995.1 mGy	(Y90) labeled microspheres	6 years (2014–2020)	TRA = 63 ± 16.5 TFA = 65 ± 17.8	TRA = 143 TFA = 45
Liu 2019	USA	Randomized controlled crossover trial	TRA = 30 TFA = 30	Left radial artery	Right common femoral artery	> 18 years old with HCC	HCC	TRA = 593.6 mGy TFA = 1,087.4 mGy	NA	6 months	TRA = 66.0 ± 12.0 TFA = 66.0 ± 12.0	TRA = 28 TFA = 28

**Table 2:** Comparison between the results of our included studies

Study ID	Loewenstern 2018	Pedersoli 2023	Bela Kis 2016	Yakupog 2023	Ghosh 2021	Liu 2019
Procedure time	NR	NR	NR	TRA = 17.8 TFA = 24.4	TRA = 111.7 TFA = 165.6	TRA = 54.9 TFA = 53.0
Recovery time	NR	NR	NR	TRA = 118 TFA = 296	NR	TRA = 108.3 TFA = 153.1
Fluoroscopy time	TRA = 9.50 TFA = 9.40	TRA = 10 TFA = 6.4	TRA = 9.45 TFA = 5.72	TRA = 10.6 TFA = 11.7	TRA = 16.1 TFA = 19.7	TRA = 13.0 TFA = 13.6
Contrast (ml)	NR	NR	NR	NR	TRA = 69.2 TFA = 75.2	NR
Dose-area product	TRA = 67,066 TFA = 67,219	NR	NR	NR	NR	TRA = 139,476 TFA = 277,949
Air Kerma	TRA = 323.63 TFA = 248.46	NR	TRA = 597.8 TFA = 302.8	TRA = 545.2 TFA = 892.7	TRA = 880.2 TFA = 995.1	TRA = 593.6 TFA = 1,087.4
Pain after the procedure	NR	NR	NR	NR	NR	NR
Pain in recovery room after procedure	NR	NR	NR	TRA = 1.67 TFA = 1.68	NR	TRA = 2.1 TFA = 2.9
Pain after discharge at home	NR	NR	NR	NR	NR	TRA = 1.4 TFA = 1.5
Pain at access site during procedure	NR	NR	NR	NR	NR	TRA = 2.0 TFA = 3.0
Pain overall during procedure	NR	NR	NR	TRA = 1.83 TFA = 1.74	NR	TRA = 2.0 TFA = 2.9
Mental component summary	NR	NR	NR	TRA = 59.27 TFA = 57.13	NR	TRA = 73 TFA = 71
Physical component summary	NR	NR	NR	TRA = 62.1 TFA = 59.3	NR	TRA = 64 TFA = 64
Adverse events	NR	NR	TRA = 0/188 TFA = 1/63	TRA = 2/75 TFA = 0/147	TRA = 3/27 TFA = 0/23	NR
Hospitalization time,	NR	NR	NR	TRA = 244 TFA = 411	NR	NR

TRA = transradial approach, TFA = transfemoral approach, NR = not reported

Assessment of study quality using the NOS indicated good quality across most studies, except Yakupoglu et al. 2023 [15], which was rated as fair (Supplementary file Fig. 1).

The ROB2 assessment of Liu et al. [16] indicated a low risk of bias in randomization, outcome measurement, and result selection. However, it highlighted concerns regarding deviations from intended interventions and missing outcome data. Overall, the study was rated as having some concern.

### Patient Radiation Exposure

#### Procedure Time

**Fig. 2:**
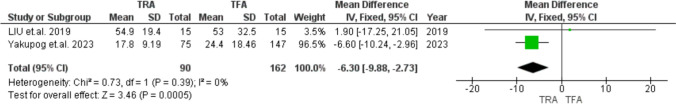
Forest plot comparing TRA versus TFA for procedure time

The TRA group showed a significantly shorter procedure time compared to the TFA group (MD = − 6.30, 95% CI [− 9.88– − 2.73], *P* = 0.005). The pooled studies were homogenous (I2 = 0%, *P* = 0.39) (Fig. 2).

#### Fluoroscopy Time

**Fig. 3:**
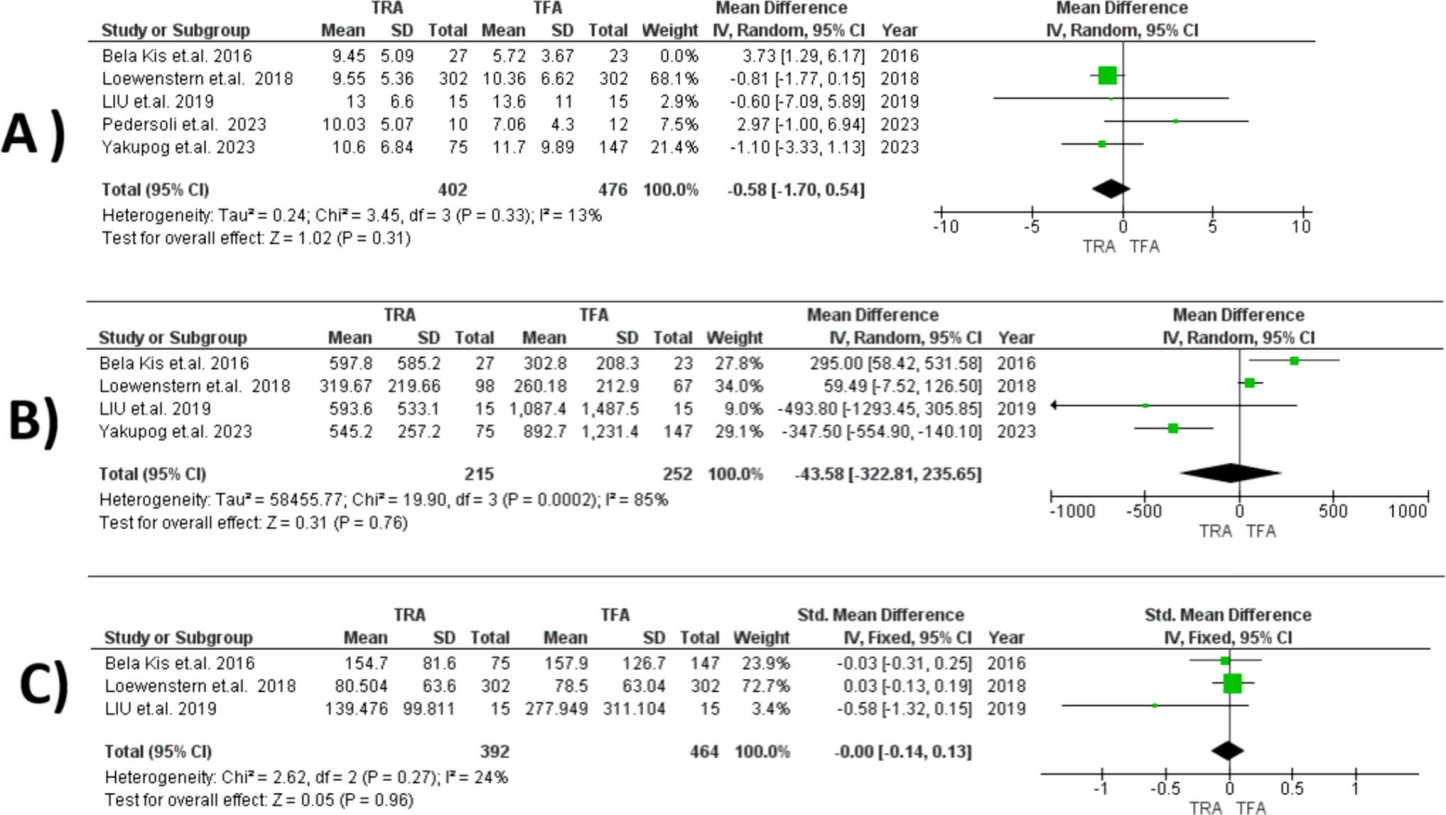
Forest plots comparing TRA versus TFA for: **A** fluoroscopy time, **B** air kerma and, **C** dose-area product

The TRA group had a nonsignificantly shorter fluoroscopy time compared to the TFA group (MD = − 0.75, 95% CI [− 0.41, 2.90], *P* = 0.50), with substantial heterogeneity (I2 = 73%, *P* = 0.006). Excluding Bela Kis et al. [3] reduced heterogeneity (I2 = 13%, *P* = 0.33), but the difference remained statistically insignificant (MD = − 0.58, 95% CI [− 1.70, 0.54], *P* = 0.31) (Fig. 3A).

#### Air Kerma

The analysis revealed no significant difference between the TRA and TFA groups [3, 14–16] (MD = − 43.58, 95% CI [− 322.81, 235.65], *P* = 0.76), with substantial heterogeneity among the studies (I2 = 85%, *P* = 0.0002). This heterogeneity could not be resolved by sensitivity analysis (Fig. 3B).

#### Dose-Area Product

The analysis revealed no significant difference between the TRA and TFA groups [3, 14, 16] (MD = 0.00, 95% CI [− 0.14, 0.13], *P* = 0.96). The analysis demonstrated homogeneity in results (I2 = 24%, *P* = 0.27) (Fig. 3C).

### Safety Outcomes

#### Overall Pain During the Procedure

**Fig. 4:**
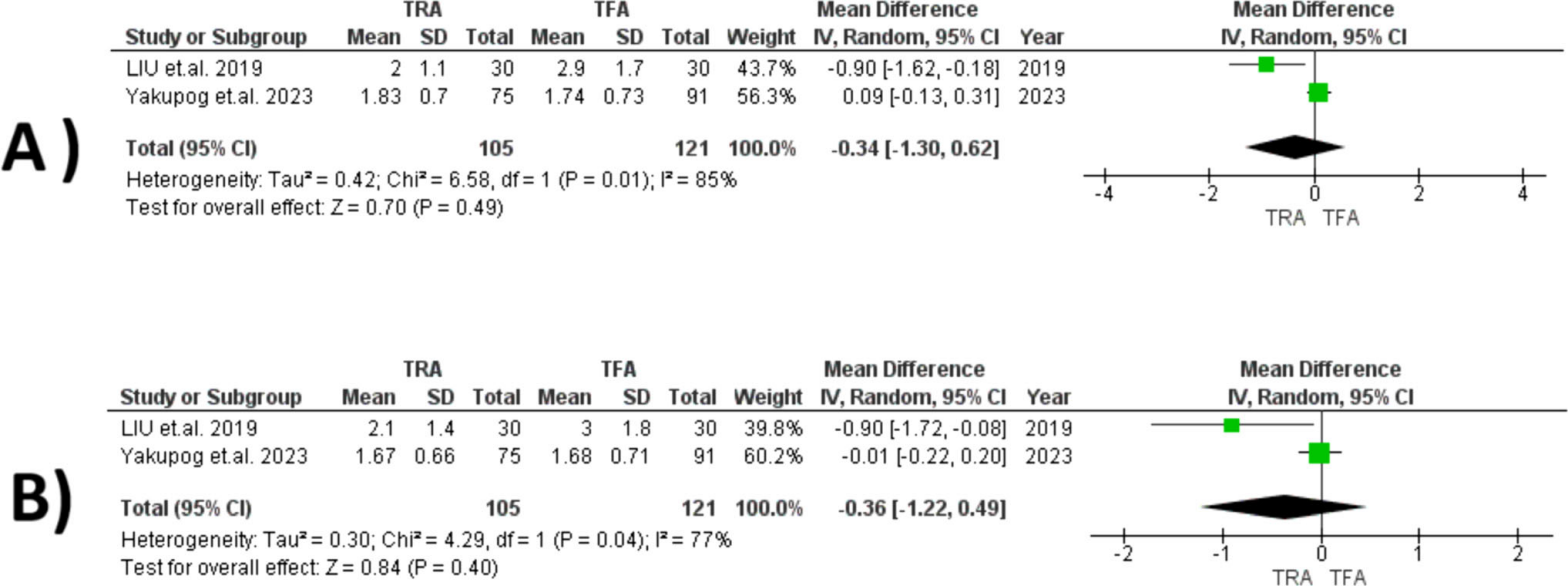
Forest plots comparing TRA versus TFA for: **A** overall pain during the procedure, and **B** pain in the recovery room after the procedure

The analysis revealed no significant difference between the TRA and TFA groups [15, 16] (MD = − 0.34, 95% CI [− 1.3, 0.62], *P* = 0.49). Despite this, considerable heterogeneity was observed (I2 = 85%, *P* = 0.01). Due to the limited number of studies, a leave-one-out test could not be performed (Fig. 4A).

#### Pain in the Recovery Room After the Procedure

The analysis revealed no significant difference between the TRA and TFA groups (MD = − 0.36, 95% CI [− 1.22, 0.49], *P* = 0.40), accompanied by substantial heterogeneity (I2 = 77%, *P* = 0.004). Due to the limited number of studies, a leave-one-out test could not be conducted (Fig. 4B).

Pain assessment outcomes, including pain after the procedure, pain after discharge at home, and pain at the access site during the procedure, were documented in Liu et al.’s [16] study and are summarized in Table 2**.**

#### Recovery Time

Our analysis found no significant difference between TRA and TFA (MD = − 112.58, 95% CI [− 243.09, 17.93], *P* = 0.09), accompanied by significant heterogeneity (I2 = 98%, *P* < 0.00001). Due to the limited number of studies, a leave-one-out test could not be conducted (Supplementary Figure S2).

#### Adverse Events 

The incidence of adverse events was slightly higher in the transradial access group (5/290, 1.7%) compared to the transfemoral access group (1/233, 0.43%). However, this difference was not statistically significant (RR = 1.98, 95% CI [0.13–29.33], *P* = 0.62), and moderate heterogeneity was observed (I2 = 58%, *P* = 0.09) [7]. This heterogeneity could be resolved by excluding Ghosh et al. [7] (I2 = 0%, *P* = 0.82). Nevertheless, the statistical insignificance of the outcome persisted (RR = 7.75, 95% CI [0.93–61.71], *P* = 0.06) (Supplementary Figure S3).

### Impact on the Quality of Life

Quality of life was assessed in two studies: Liu et al. [16] and Yakupog et al. [15]. However, Liu et al. [16] utilized the 12-item Short Form Health Survey, which comprises the Physical Component Summary and Mental Component Summary. In contrast, Yakupog et al. [15] employed the 36-item Short-Form Health Questionnaire, measuring mental health and physical function.

#### Mental Health

**Fig. 5:**
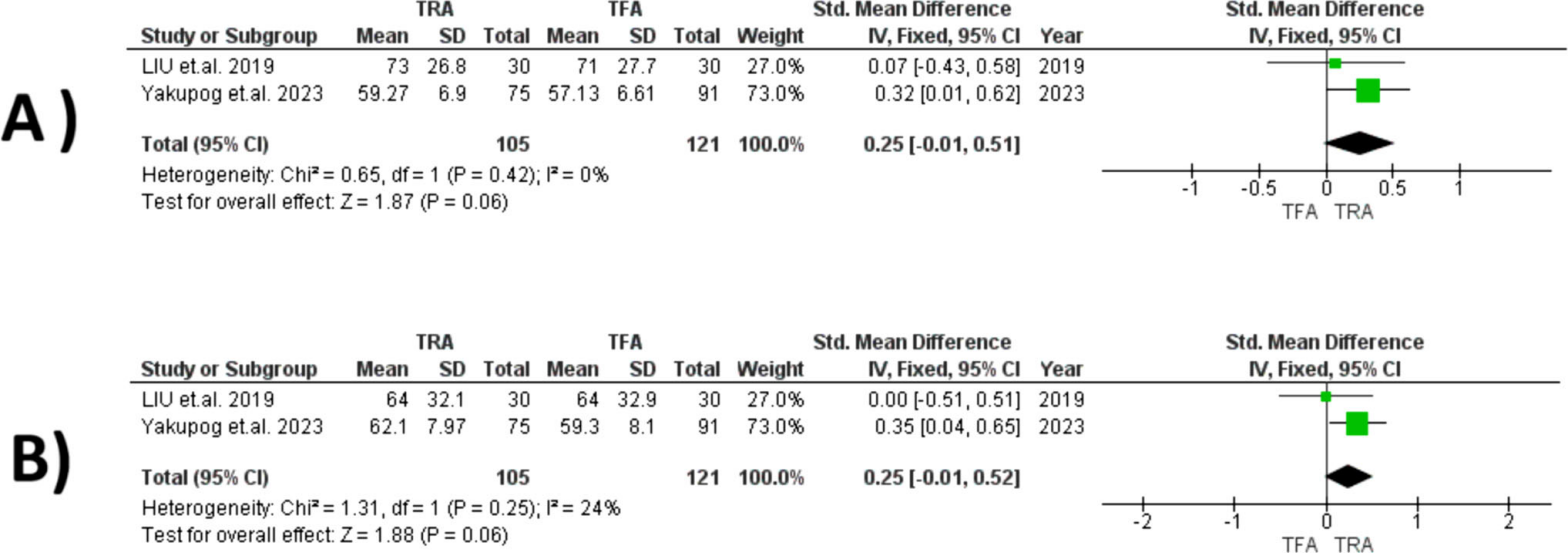
Forest plots comparing TRA versus TFA for: **A** mental health, and **B** physical function

Our analysis indicated no significant difference between TRA and TFA (SMD = 0.25, 95% CI [− 0.01, 0.51], *P* = 0.06), with significant homogeneity observed among the studies (*I*^2^ = 0%, *P* = 0.42) (Fig. 5A).

#### Physical Function

Our analysis indicated no significant difference between TRA and TFA (SMD = 0.25, 95% CI [− 0.01, 0.52], *P* = 0.06), with significant homogeneity observed among the studies (*I*^2^ = 24%, *P* = 0.25). (Fig. 5B).

## Discussion

### Summary of Our Findings

Our findings suggest that the procedure time for TARE is significantly shorter with TRA compared to TFA. However, this conclusion should be approached with caution, as it is based on data from only two studies. Differences between the two approaches in terms of fluoroscopy time, air kerma, DAP, post-procedure pain in the recovery room, overall procedural pain, quality of life, and adverse events were statistically insignificant.

### Explanation of Our Findings

TRA has emerged as a promising alternative to TFA for various liver interventions, such as TACE and TARE for treating both primary and secondary hepatic tumors [6, 15, 17]. While TFA has traditionally been the preferred route for hepatic radioembolization, the success of TRA in coronary interventions demonstrating fewer access-related complications, greater patient satisfaction, and lower costs suggests its potential benefits for liver procedures as well [18–20].

However, there is a lack of high-quality evidence comparing TRA and TFA for non-coronary procedures. The existing literature, largely based on retrospective analyses, includes studies on TRA for TACE [17], TARE [16, 21], uterine fibroid embolization [22], prostatic artery embolization [23], and renal artery interventions [24]. These studies consistently demonstrate high technical success rates, shorter post-procedure recovery, shorter ambulation times, reduced bleeding risks, and improved patient comfort and satisfaction with TRA, similar to the benefits observed in coronary interventions.

Patient preferences and satisfaction with medical procedures are increasingly becoming the focus of research. In a retrospective study by Horn et al. (2016), among 67 patients who underwent both transradial access (TRA) and transfemoral access (TFA) for various procedures, 53 (79%) preferred TRA, while only 4 (6%) favored TFA, with 10 (15%) expressing no preference [25]. Similarly, a prospective study by Satti et al. [26] revealed that 24 out of 25 patients (96%) preferred radial access for cerebrovascular procedures. Furthermore, in a prospective study by Yamada et al., 29 of 36 patients (81%) opted for radial access over femoral access for liver cancer radioembolization [27]. However, despite these preferences, these studies did measure specific reasons influencing patient choices, such as pain, recovery time, or post-procedural quality of life.

Liu et al. (2019) investigated patient preferences and objectively assessed the impact of access sites on comfort and satisfaction during liver cancer radioembolization. Their study included patients with hepatocellular carcinoma who had experienced both TRA and TFA procedures. The findings revealed a strong preference for TRA, with 73.3% favoring radial access compared to 13.3% preferring femoral access and 13.3% having no preference. This preference likely stems from the study’s observation of significantly less pain with TRA during both the procedure and recovery. Additionally, TRA was associated with shorter recovery times, further contributing to patient satisfaction [16]. These findings highlight the importance of considering patient preferences when selecting the approach for hepatic interventions.

Current evidence regarding radiation exposure between TRA and TFA is controversial [16, 27], while large randomized trials in interventional cardiology have not found significant differences in patient radiation exposure measures, including fluoroscopy time, DAP, and air kerma, between TRA and TFA cardiac catheterization procedures [28, 29]. Similarly, studies on patient radiation exposure in non-coronary interventions, such as transarterial chemoembolization and prostatic artery embolization, have also found no differences in FT, DAP, or AK between the TRA and TFA approaches [14]. However, some large studies and meta-analyses in coronary interventions suggest that TRA is associated with higher radiation exposure compared with TFA [30, 31]. Although various technical parameters, such as protective devices and dose-reducing techniques [7], can help mitigate operator exposure during interventions, the impact of the vascular access site on operator radiation exposure remains unclear. This uncertainty persists even as more proceduralists begin to adopt TRA for complex vascular interventions.

Our meta-analysis addressed this conflict specifically focused on hepatic tumor radioembolization. The results are encouraging, demonstrating no significant differences in fluoroscopy time, air kerma, and DAP between TRA and TFA groups. This suggests comparable radiation exposure risk during radioembolization for patients with hepatic tumors by either access route.

Interestingly, our study revealed a contrast between the procedure time and fluoroscopy time. TRA showed significantly shorter procedure times but no significant difference in fluoroscopy time compared to TFA, this can be attributed to several factors. The overall procedure time includes both fluoroscopic and non-fluoroscopic components, and TRA may have more efficient non-fluoroscopic preparation and completion processes, leading to a shorter total procedure time despite similar fluoroscopy times. Additionally, operator experience with TRA, quicker vascular access and catheter manipulation, and the efficiency of support staff can streamline the overall procedure. However, the complexity of the procedures and the fluoroscopy usage itself remain comparable across both access routes, resulting in similar fluoroscopy times.

Furthermore, this disparity may be also explained by the smaller number of studies included in the analysis of procedure time, which comprised only two studies, compared to the analysis of fluoroscopy time, which included five studies. This difference in the number of studies could affect the statistical power and the robustness of the findings.

### Comparison with Previous Research

Regarding procedure time, Liu et al. [16] found no significant difference between TRA and TFA, which contrasts with our findings. However, Yakupog et al. [15] found a significant decrease with TRA, aligning with our results.

In terms of fluoroscopy time, studies by Bela Kis et al. [3], Loewenstern et al. [14], Liu et al. [16], Pedersoli et al. [6], and Yakupog et al. [15] all found no significant difference between TRA and TFA, which aligns with our findings.

For dose-area product (DAP), Bela Kis et al. [3], Liu et al. [16], and Loewenstern et al. [14] reported no significant difference between TRA and TFA, consistent with our results.

In the air kerma analysis, Bela Kis et al. [3] found a significant increase with TRA, while Yakupog et al. [15] found a significant decrease, contrasting with our findings. Liu et al. [16] and Loewenstern et al. [14] reported no significant difference, which aligns with our results.

Regarding overall pain during the procedure and pain in the recovery room, Liu et al. [16] found a significant decrease with TRA, contrasting with our findings. Yakupog et al. [15] found no significant difference, which aligns with our results.

For recovery time, Yakupog et al. [15] and Liu et al. [16] found a significant decrease with TRA, contrasting with our findings. Lastly, in terms of adverse events, Bela Kis et al. [3] and Yakupog et al. [15] found no significant difference between TRA and TFA, which aligns with our results.

### Strengths, Limitations, and Future Research Directions

The strength of our study lies in its comprehensive evaluation of TARE procedures for liver cancer treatment, with a comparative analysis of the TRA and TFA approaches. By synthesizing data from six included studies, which encompassed both retrospective cohort analyses and a RCT, and included a total of 1209 patients, we conducted a thorough examination of these interventions. Assessing 10 distinct outcomes, including patient radiation exposure, quality of life, and safety parameters, our study provided a robust analysis to determine which approach offers superiority in improving patient outcomes. Additionally, the focus on one hepatic intervention provides solid ground for clinical guidelines.

However, our study faced limitations due to the small number of included studies and variations in study design and patient populations, which could introduce biases and affect result generalizability. We encountered heterogeneity in some analyses and did not assess outcomes such as pain after the procedure, pain at home, or pain at the access site and perform cost analysis, as these were not reported in the studies. Additionally, some analyses were based on data from only two studies, which may impact the generalizability of our findings.

To address these limitations and advance the field of TARE for liver cancer, future research should focus on conducting well-designed RCTs with larger sample sizes to validate and strengthen our results. Studies should explore long-term outcomes, including tumor response rates, overall survival, and quality of life with different access approaches (transradial versus transfemoral). Investigating predictors of adverse events and patient outcomes could enhance patient selection and procedural planning. Furthermore, comparative studies on the cost-effectiveness of various access approaches would be valuable for healthcare decision-making. Continued research is essential to refine clinical practices and improve patient outcomes in TARE interventions.

## Conclusion

In conclusion, our meta-analysis indicates that both the transradial and transfemoral access routes exhibit comparable results in the context of transarterial radioembolization for liver tumors. Notably, transradial access offers the advantage of significantly shorter procedure duration, which may have practical implications in clinical practice. However, this finding should be considered with caution, given the analysis was based on data from only two studies. Future studies should focus on validating these findings in larger and more diverse populations, examining long-term outcomes, and exploring cost-effectiveness to further guide clinical practice and improve patient care.

## Supplementary Information

Below is the link to the electronic supplementary material.

Supplementary file1 (DOCX 347 KB)

## Data Availability

All data generated or analyzed during this study are included in this published article or the data repositories listed in the references.
