# Supplementary material for: Comparative Analysis of Transradial and Transfemoral Approaches in Transarterial Radioembolization for Liver Tumors: A Systematic Review and Meta-Analysis
Source: Cardiovasc Intervent Radiol. 2024 Oct 7;47(12):1696–707. doi: 10.1007/s00270-024-03865-z (PMC11621202; doi:10.1007/s00270-024-03865-z)

**Comparative Analysis of Transradial and Transfemoral Approaches in Transarterial Radioembolization for Liver Tumors: A Systematic Review and Meta-Analysis**

**Hatem Abdelmoneim Eldeeb ^1^,**  **Mahmoud Shaaban Abdelgalil ^2^* ,Asem Ahmed Ghalwash^3, 4^,** **Asmaa Elganady^5^ ,Ruaa Mustafa Qafesha^6^ , Ibraheem M alkhawaldeh^7^, Mahmoud Diaa Hindawi^8^, Jaber H. Jaradat^9^ , Shabaan Mohamed Abduljalil^10^,Hussien Ahmed H. Abdelgawad ^11,4^**

1. Faculty of Medicine, Alazhar University, Cairo, Egypt.

[H.eldeeb962@gmail.com](mailto:H.eldeeb962@gmail.com)

ORCID: [**https://orcid.org/0000-0001-6270-3180**](https://orcid.org/0000-0001-6270-3180)

1. Faculty of Medicine, Ain-Shams University, Cairo, Egypt

29908068800596@med.asu.edu.eg

ORCID ID: 0000-0002-7325-0129

1. Faculty of Medicine, Al Azhar University, Cairo, Egypt

[Assimghalwash0@gmail.com](mailto:Assimghalwash0@gmail.com)

ORCID: [**https://orcid.org/0009-0004-0898-6764**](https://orcid.org/0009-0004-0898-6764)

1. Medical Research Group of Egypt, Negida Academy, Arlington, MA, United States.
2. Faculty of Medicine, Alexandria University, Egypt

[asmaa.elganady@gmail.com](mailto:%20%20%20%20%20%20%20%20asmaa.elganady@gmail.com)

ORCID ID : [**https://orcid.org/0000-0001-9717-4688**](https://orcid.org/0000-0001-9717-4688)

1. Faculty of Medicine, Al-Quds University, Jerusalem, Palestine

[dr.ruaamustafa@gmail.com](mailto:dr.ruaamustafa@gmail.com)

ORCID ID: [**https://orcid.org/0000-0002-8745-0260**](https://orcid.org/0000-0002-8745-0260)

1. Faculty of Medicine, Al Azhar University, Cairo, Egypt

[dr.md.hunt@gmail.com](mailto:dr.md.hunt@gmail.com)

ORCID ID: [**https://orcid.org/0009-0001-3085-6665**](https://orcid.org/0009-0001-3085-6665)

1. Faculty of Medicine, Mutah University, Al-Karak-Jordan

[Ibraheemfamous096@gmail.com](mailto:Ibraheemfamous096@gmail.com)

ORCID ID : <https://orcid.org/0000-0002-0187-1583>

1. Faculty of Medicine, Mutah University, Al-Karak, Jordan

[jaberjaradat2002@gmail.com](mailto:jaberjaradat2002@gmail.com)

ORCID ID : <https://orcid.org/0000-0002-6488-4664>

1. Eradah complex and Mental Health hospital ,Najran, Kingdom of Saudi Arabia

[drshabanmohamed@yahoo.com](mailto:drshabanmohamed@yahoo.com)

11.Department of Child Health, University of Arizona College of Medicine, Phoenix, AZ, USA

habdelgawad@phoenixchildrens.com

ORCID ID : https://orcid.org/0000-0001-7101-837X

**Corresponding author*:**

Faculty of Medicine, Ain-shams University, Cairo, Egypt.

Postal address; 359 Abd Allah Nadim Street, Cairo, Egypt.

Email: 29908068800596@med.asu.edu.eg

ORCID: 0000-0002-7325-0129

Phone: (+20)1154257518

**Search strategy: -**

**PubMed;**

(transradial OR TRA OR trans-radial OR Radial access OR Radial artery access) AND (Femoral artery access OR femoral access OR transfemoral OR trans-femoral OR TFA) AND (Radioembolization, Therapeutic OR radioembolization OR radioembolisation OR TARE OR hepatic arterial infusion radiotherapy OR Transcatheter arterial radioembolization OR transcatheter radioembolization OR TAE OR transarterial embolization)

No limitations were applied.

Filed of search: All fields’

From inception to October 8, 2023

Results: **25**

**Cochrane:**

(transradial OR TRA OR trans-radial OR Radial access OR Radial artery access) AND (Femoral artery access OR femoral access OR transfemoral OR trans-femoral OR TFA) AND (Radioembolization, Therapeutic OR radioembolization OR radioembolisation OR TARE OR hepatic arterial infusion radiotherapy OR Transcatheter arterial radioembolization OR transcatheter radioembolization OR TAE OR transarterial embolization)

No limitations were applied.

From inception to October 8, 2023

Filed of search: All fields

Results: **4**

**Web of Science:**

("transradial" OR "TRA" OR "trans-radial" OR "Radial access" OR "Radial artery access") AND

("Femoral artery access" OR "femoral access" OR "transfemoral" OR "trans-femoral" OR "TFA") AND

("Radioembolization, Therapeutic" OR "radioembolization" OR "radioembolisation" OR "TARE" OR "hepatic arterial infusion radiotherapy" OR

"Transcatheter arterial radioembolization" OR "transcatheter radioembolization" OR "TAE" OR "transarterial embolization")

No limitations were applied.

From inception to October 8, 2023

Results: **26**

**Scopus:**

("transradial" OR "TRA" OR "trans-radial" OR "Radial access" OR "Radial artery access") AND

("Femoral artery access" OR "femoral access" OR "transfemoral" OR "trans-femoral" OR "TFA") AND

("Radioembolization, Therapeutic" OR "radioembolization" OR "radioembolisation" OR "TARE" OR "hepatic arterial infusion radiotherapy" OR

"Transcatheter arterial radioembolization" OR "transcatheter radioembolization" OR "TAE" OR "transarterial embolization") No limitations were applied.

From inception to October 8, 2023

Results: **19**

**EMBASE:**

(transradial OR TRA OR trans-radial OR Radial access OR Radial artery access) AND (Femoral artery access OR femoral access OR transfemoral OR trans-femoral OR TFA) AND (Radioembolization, Therapeutic OR radioembolization OR radioembolization OR TARE OR hepatic arterial infusion radiotherapy OR Transcatheter arterial radioembolization OR transcatheter radioembolization OR TAE OR transarterial embolization)No limitations were applied.

From inception to October 8, 2023

Results: **35**

**Supplementary Figure S1: Newcastle-Ottawa Scale Assessment for Retrospective Cohort Studies**


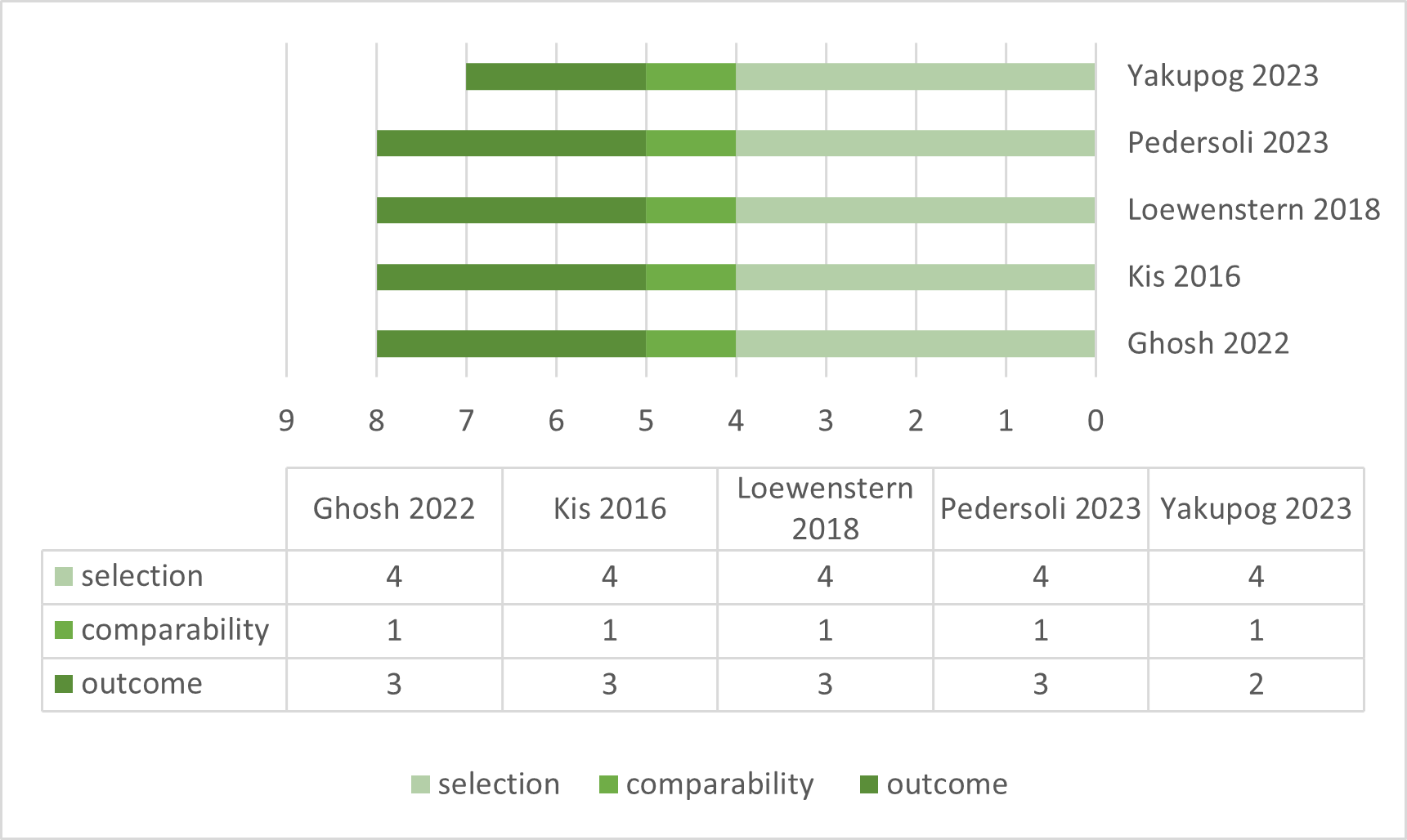


**Supplementary Figure S2: Forest plot comparing TRA versus TFA for Recovery time**


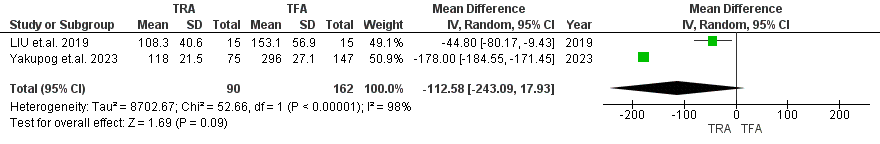


**Supplementary Figure S3. Forest plot comparing TRA versus TFA for Adverse Events**


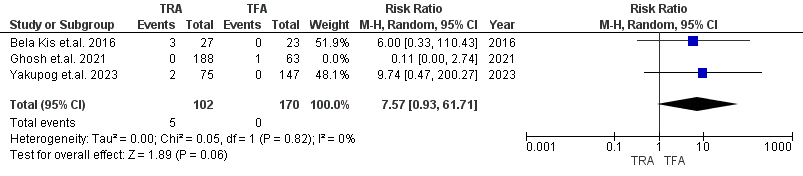

Supplement: Supplementary file 1 — Supplementary file1 (DOCX 347 KB) [file 270_2024_3865_MOESM1_ESM.docx]
